# The feasibility of mindfulness-based cognitive therapy for people with bipolar disorder: a qualitative study

**DOI:** 10.1186/s40345-020-00197-y

**Published:** 2020-11-11

**Authors:** Imke Hanssen, Nicole van der Horst, Marieke Boele, Marc Lochmann van Bennekom, Eline Regeer, Anne Speckens

**Affiliations:** 1 Radboud University Medical Centre, Department of Psychiatry, Centre for Mindfulness, Postbus 9101, 6500 HB Nijmegen, The Netherlands; 2 Donders Institute for Brain, Cognition and Behaviour, Radboud University, Nijmegen, The Netherlands; 3 Pro Persona Institute for Mental Health Care, Outpatient Clinic for Bipolar Disorders, Tarweweg 2, 6534 AM Nijmegen, The Netherlands; 4 Altecht Institute for Mental Health Care, Outpatient Clinic for Bipolar Disorder, Lange Nieuwstraat 119, 3512 PG Utrecht, The Netherlands

**Keywords:** MBCT, Bipolar disorder, Barriers, Facilitators, Qualitative research

## Abstract

**Background:**

Mindfulness- Based Cognitive Therapy (MBCT) could be a promising psychosocial intervention for people with bipolar disorder (BD). However, little is known about the feasibility of MBCT for people with BD. In this study we explore the facilitators and barriers people with BD experience of an adapted MBCT program.

**Method:**

This qualitative study is part of a large, multicenter randomized controlled trial on MBCT for BD (trial registration number: NCT03507647). The present study included 16 participants with BD who participated in an 8-week adapted MBCT program. Semi- structured interviews exploring the feasibility, with a particular focus on the bipolar symptoms, were recorded verbatim, transcribed and analyzed. For reasons of triangulation, teachers were interviewed as well.

**Results:**

Participants reported different barriers and facilitators of MBCT, both generally as well as with regard to their bipolar disorder. Four key themes arose: the training itself, psychosocial factors, personal characteristics and the bipolar disorder. Themes were further divided in subthemes.

**Conclusion:**

The adapted MBCT program seemed to be feasible for people with BD. Depressive symptoms often acted as a barrier for participating in MBCT, suggesting that participants might need additional support when depressed. Manic symptoms could act both as a barrier and facilitator, suggesting that the occurrence of (hypo)mania does not necessarily have to be an exclusion criterion for participation. Further clinical and research implications are suggested.

Trial registration: ClinicalTrials.gov, NCT03507647. Registered 25th of April 2018, https://clinicaltrials.gov/ct2/show/NCT03507647.

## Background

Bipolar disorder (BD) is a severe and chronic mood disorder with patients suffering from recurrent depressive, (hypo) manic and/or mixed episodes. In the Netherlands, the prevalence of BD is around 1.2% of men and 1.4% of woman (De Graaf et al. 2010). BD is associated with high relapse, morbidity and mortality rates (Novick et al. 2010), and accounts for equally high or even higher levels of impairments and disabilities as compared to major depression and schizophrenia (Pini et al. 2005). Among the mental and substance use disorders assessed in the Global Burden Disease Study in 2013 (Ferrari et al. 2016), BD was the fifth leading cause of daily adjusted life years and is associated with high levels of psychological stressors, like internalized stigma, subjective distress (Davis and Kurzban 2012), self- blame (Valtonen et al. 2007), suicidal ideation (Valtonen et al. 2005), and a lower quality of life (Pascual-Sánchez et al. 2019). Pharmacological interventions have been the first- line treatment of BD. However, even with medication 60% of the patients relapse within 2 years (Miklowitz et al. 2009). Besides existing psychosocial interventions, there is a growing need for additional interventions to help people with BD to cope with their illness, to reduce the risk of relapse and to improve social and psychological wellbeing and quality of life (Farkas 2007).

Mindfulness- Based Cognitive Therapy (MBCT) could be a promising psychosocial intervention for BD by means of reducing residual depressive symptoms, attentional difficulties, emotional-regulation problems and to improve mindfulness, psychological well-being, positive affect and psychosocial functioning (Deckersbach et al. 2012). It involves practicing to be present ‘in the moment’ and an attitude of non- judgmental acceptance with the aim to maintain awareness, disengaging oneself from strong attachment and thereby developing a greater sense of emotional balance and well-being (Kabat-Zinn 2013). MBCT was developed to reduce relapse in people with a history of major depressive disorder (Segal et al. 2002) and has showed efficacy in reducing the risk of relapse and recurrence (Kuyken et al. 2016). MBCT is considered to decrease relapse by enhancing recognition, decentering and disengaging from ruminative thought patterns, developing meta- awareness and fostering self-compassion (Segal et al. 2012). Besides, meditation had been shown to strengthen executive functions (Chiesa et al. 2011). Furthermore, MBCT has been shown to be effective in other disorders as well, such as current depression, pain conditions, smoking, addictive disorders, anxiety, and stress (Goldberg et al. 2018). Moreover, it appears to be promising in helping patients cope with severe mental illness, such as BD (Davis and Kurzban 2012; Lovas and Schuman-Olivier 2018).

Mindfulness in people with BD has been increasingly studied over the past few years, with promising results (Miklowitz et al. 2009; Deckersbach et al. 2012; Weber et al. 2010). A recent systematic review has demonstrated a positive effect of MBCT on anxiety, residual depressive symptoms, mood regulation, and reduction in manic symptoms in people with BD (Lovas and Schuman-Olivier 2018). Except for one study all included studies in that review used an 8-week MBCT protocol with only minor adaptations to BD (e.g. adding psychoeducation on BD). One study used an 12-week MCBT program with major adaptations (e.g. including loving kindness meditation, more movement exercises, problem solving skills). (Deckersbach et al. 2012).More high-quality studies are needed to underpin the clinical efficacy in this population, as most of the studies included in the review were underpowered and of moderate quality.

Despite increasing research in the efficacy of MBCT in BD, there are only a few studies that write about the feasibility and the barriers and facilitators people with BD experience when participating in MBCT. To our knowledge, there is only one open study trial on the feasibility of MBCT for BD (Weber et al. 2010), and one study about the experiences of mindfulness practices of people with BD (Chadwick et al. 2011). The study of Weber and colleagues (Weber et al. 2010) included 23 participants by whom feasibility was assessed with self-rated questionnaires. They conclude that MBCT seems feasible and well-perceived among people with BD. However, the study reports a drop-out rate of 35%, and the underlying reasons for drop-out remain unclear. Furthermore, barriers of MCBT were not investigated and it is uncertain whether symptoms of BD influenced the experience and attendance of the training. Finally, the MBCT offered in the Weber study consisted of 8 weekly sessions of 2 h. In clinical practice MBCT sessions are often prolonged to 2.5 h, and include a 6 h silent day between session 6 and 7 (Witkiewitz 2011). The study of Chadwick and colleagues (Chadwick et al. 2011) used qualitative methodology to explore the experiences of 12 people with BD who participated in an adapted, shorter version of the original MBCT-program. They concerned on the process and general experience of MBCT for BD, and did not specifically focus on feasibility. Nevertheless, they suggest the need to consider changes to MBCT for people with BD, such as more flexible use of homework, shorter sessions and meditation practices, and a greater emphasize on integrating mindfulness in daily life. However, the MBCT offered in the Chadwick study was substantially adapted: the sessions were much shorter (weekly sessions of 90 rather dan 120–150 min) and meditations briefer (10 rather than 45 min). Therefore, it might be of interest to qualitatively investigate the experiences of people with BD when participating in MBCT with only minor adaptations to BD. Based on the findings, the MBCT protocol could be adapted to the needs of people with BD.

The aim of the present qualitative study is to specifically address which barriers and facilitators people with BD experience when participating in MBCT. This study is part of a large, multicenter randomized controlled trial (RCT) on the (cost-)effectiveness of MBCT for BD (Hanssen et al. 2019).

## Methods

### Participants

The subjects of the present study were participants in a large, prospective, evaluator-blinded RCT on the (cost-)effectiveness of MBCT in people with BD in the Netherlands (Hanssen et al. 2019). Participants were included in the RCT when they met the following criteria: (1) a diagnosis of bipolar I or II disorder (as assessed with the Structural Clinical Interview DSM-5 (First et al. 2016)); (2) at least two lifetime depressive episodes, either current or in (partial) remission at baseline (as assessed with the Inventory of Depressive Symptomatology—Clinician Rated (IDS-C) (Trivedi et al. 2004); 3) at least one affective episode (depressed or (hypo)manic) within the year prior to baseline; and 4) score < 12 on the Young Mania Rating Scale (YMRS) (Young et al. 2000). Exclusion criteria were: (1) a (hypo)manic episode within 3 months before the start of the trial; (2) a lifetime diagnosis of schizophrenia or schizoaffective disorder; (3) current substance abuse disorder; (4) antisocial or borderline personality disorder; (5) risk of suicide or aggression; and (6) the presence of a concurrent significant medical condition impeding the ability to participate.

Unlike quantitative research, which only gives a surface description of a larger sample, qualitative research is aimed at gaining a deeper understanding of a phenomenon from the perspective of the participants (Silverman et al. 2013). Therefore, participants are included by means of purposive sampling. With purposive sampling, participants are selected based on important criteria in order to gain as much insight from as many different angles as possible. (The SAGE 2018; Palinkas et al. 2015). Criteria which were used to purposively sample participants in the current study were the following: gender, age, diagnoses (BD type I or II), participating study sites (Pro Persona, Dimence and Altrecht), and drop-out versus non drop-out. Data collection did not stop before data saturation was reached. In qualitative research, data saturation occurs when new data repeat what was expressed in previous data, in which case further data collection becomes counter-productive and unnecessary (Saunders et al. 2018). In the current study data saturation was reached after interviewing 16 participants.

### Intervention

The training was based on MBCT for recurrent depression, a manualized group skill training program designed as a relapsed prevention program (Segal et al. 2012). The training consisted of 8 weekly sessions of 2.5 h, plus one silent day. In addition, participants were instructed to practice 45 min a day with guided mindfulness exercises and to complete homework assignments in a workbook. The training was adapted to the needs of people with BD in the sense that psycho-education about (hypo)manic and depressive symptoms was included, the 3- min breathing space was introduced earlier and more often in the program, possible partners were involved in session six and movement exercises were more frequently used (Hanssen et al. 2019). These adaptations were the result of the outcome of two earlier focus groups of 15 people with BD who participated in traditional MBCT at the Radboud University Medical Centre (Nijmegen, the Netherlands).

MBCT was taught by pairs of one qualified MBCT teacher and one nurse specialized in the care of people with BD. The MBCT teacher met the advanced criteria of the Association of Mindfulness Based Teachers in the Netherlands and Flanders and the internationally agreed good practice guidelines of the UK Network for Mindfulness- Based Teachers (Hanssen et al. 2019; Crane et al. 2012). Each group comprised 8–10 participants. Participants received the MBCT in addition to treatment as usual (TAU) by their own practitioners, consisting of psycho-education, medication and self-management.

### Procedure

Participants of the RCT were recruited in six participating outpatient clinics specialized in BD. They received a letter from their attending clinicians, informing them about the study with an included information leaflet. Interested patients were screened to assess eligibility, and informed consent was acquired. Eligible patients were randomized to either the intervention group (MBCT + TAU) or control group (TAU). Participants did not receive any payment for participation. Please see Hanssen et al. 2019 for more extensive information about the procedure.

Within 3 months after completing MBCT, participants of three specialized outpatient clinics (Pro Persona, Altrecht, Dimence) were invited by telephone to take part in an individual face to face interview about their experienced barriers and facilitators of MBCT. Before starting the interviews, the Altman Self-Rating Mania Scale (ASRM) (Altman et al. 1997) and the Quick Inventory of Depressive Symptomatology—Self Report (QIDS-SR) (Rush et al. 2003) were administered to screen for the presence of (hypo) manic and depressive symptoms. A score of ≥ 6 on the ASRM and a score of ≥ 25 on the QIDS-SR was regarded as a contra-indication for attending the interview. The interviews were conducted by two-third-year female residents in psychiatry (NH, MB). The interviewers had not been involved in the clinical care or MBCT training of the participants, but had participated in an MBCT training themselves. One of them was experienced in meditation. Before starting the interviews, interviewers introduced themselves, their occupation and their role in the study to the participants. After this, the interviewers asked permission to record the interview. During the interview one of the interviewers was in the lead, the other interviewer made key notes, asked some additional questions and monitored the bigger picture throughout the interview. Most interviews were conducted in the outpatient clinic, one interview was conducted at the participants’ home because of the travelling distance. The interviews took 45 to 90 min.

### Topic guide

The semi-structured interview consisted of a topic list that was constructed to address the main question: what were facilitators and barriers during MBCT, generally and with regard to bipolar disorder specifically. The interview started with two open-ended questions: ‘*In your experience, what things made it easier for you to attend the training?*’ and ‘*What things made it more difficult for you to attend the training?*’ Furthermore, the topic list included prompts that specifically asked about topics that were still underexposed. During the interview, the interviewers would probe for more detail when deemed necessary. Interviews were recorded with a Dictaphone, transcribed verbatim, sent to the participants for verification, and adapted in case of any corrections from the participants.

### Qualitative analysis

All semi- structured interviews were coded and analyzed by the interviewers in a multistage process, using the methodological orientation of a grounded theory (Boeije 2014). The research team consisted of six people: three psychiatrists, one research psychologist and two residents in psychiatry. Two psychiatrists of the team had special expertise in diagnosing and treating adults with BD (MLvB and ER), one in the application of mindfulness-based interventions (AS). Two members of the team were familiar with qualitative research (ER and AS). The qualitative analysis consisted of three coding phases, namely open coding, axial coding and selective coding (Boeije 2014). In this way, coding was data driven instead of theory driven. A repeating cycle was used, which consisted of conducting five interviews, transcribing and coding these five interviews, and analyzing the data with the research team. After this, it was determined which information remained underexposed, as a result of which additional questions were added to the topic guide. Examples of topics that were added to the topic list were for example: the silent day, partner session (session six), influence of mood symptoms on mindfulness practice, and the group setting. Then the next five interviews were conducted. Open coding consisted of reading and re-reading the interviews and developing a code tree. During the first interviews this was conducted by both interviewers together. To ensure reliability, transcripts were coded independently by the two interviewers and the codes were compared and discussed until agreement was reached. Codes were also discussed in the research team. The list of codes was used during the coding of the next interviews. This was followed by the next phase of axial coding in which the interviewers made a list of categories and (sub) themes by the list of codes, which was discussed with the research team. After this phase, the findings were integrated within the different categories and checked and re-integrated by the research team. During this process the program Atlas.ti, software was used for analyzing and classifying of qualitative data (Ti 2019).

For reasons of triangulation, the six teachers and nurses who provided the training were interviewed as well, by means of a focus group. The focus group was transcribed and coded by one researcher (IH) who was part of the research team.

## Results

### Study population

Sociodemographic and clinical characteristics of the participants are provided in Table 1. Nineteen participants were invited for the present study, of whom three were not willing to participate because of lack of time. None of the participants scored above the cut-off levels of the ASRM (score ≥ 6) and the QIDS-SR (score ≥ 25). All participants in the current study used mood stabilizing medication. Three participants also used concurrent benzodiazepines to relieve anxiety symptoms.

**Table 1:** Sociodemographic and clinical characteristics

ID	Gender	Age	Higher education	Study site	Type of BD	Duration of illness (in years)	Total number of mood episodes	Mood Stabilizing medication (MS) and/or Benzodiazepines (B)	YMRS score baseline	IDS-C score baseline	Total number of MBCT sessions^a^	Drop-out^b^	ASRM score during interview
1	Male	47	Yes	Altrecht	Type I	31	52	MS	0	5	2	Yes	1
2	Male	65	Yes	Altrecht	Type II	35	130	MS	4	8	7	No	5
3	Male	47	Yes	Altrecht	Type II	31	61	MS	0	20	9	No	1
4	Male	69	No	Pro Persona	Type I	13	8	MS	4	4	3	Yes	2
5	Female	51	Yes	Pro Persona	Type I	33	7	MS	4	10	7	No	1
6	Male	49	Yes	Pro Persona	Type II	22	4	MS	1	6	8	No	2
7	Female	45	No	Pro Persona	Type I	23	5	MS	2	16	6	No	4
8	Female	24	Yes	Pro Persona	Type I	3	5	MS	2	27	7	No	0
9	Male	53	Yes	Altrecht	Type I	25	27	MS	0	53	9	No	0
10	Female	54	No	Altrecht	Type II	12	4	MS	2	3	6	No	1
11	Female	71	No	Dimence	Type II	45	12	MS	0	8	3	Yes	0
12	Female	55	No	Altrecht	Type I	25	70	MS + B	3	18	9	No	3
13	Male	30	Yes	Altrecht	Type II	7	16	MS	1	0	9	No	0
14	Female	64	No	Altrecht	Type I	14	35	MS + B	9	14	9	No	1
15	Female	58	Yes	Altrecht	Type I	40	60	MS	0	14	9	No	5
16	Male	66	Yes	Altrecht	Type I	47	9	MS + B	4	51	8	No	3

^a^Including silent day

^b^Drop-out yes = attended less than 4 MBCT sessions

### Facilitators and barriers

Of the 16 included participants, three (19%) attended less than four sessions of MBCT and were considered as drop-outs. Reasons for drop-out were relapse into mania (*n *= 2) and not being able to attend MBCT because of work (*n* = 1). Analysis of the data revealed four broad categories of perceived facilitators and barriers: (De Graaf et al. 2010) *training,* (Novick et al. 2010) *psychosocial factors,* (Pini et al. 2005) *personal characteristics and* (Ferrari et al. 2016) *bipolar disorder*. Each of the broad categories were subdivided in themes. Two themes were further subdivided into subthemes. Most of the (sub) themes could act as both facilitator and barrier, only a few (sub)themes acted as barrier only (see Table 2). Citations illustrating the (sub) themes are shown in Table 3.

**Table 2:** Themes and subthemes of facilitators and barriers during MBCT

Barriers	Facilitators
Training	Training
Setting	Setting
Location	Location
Space	Space
Time	Time
Content	Content
Physical content	Physical content
Materials	Materials
Homework	Homework
Teacher	Teacher
Peer group	Peer group
1. Psychosocial	1. Psychosocial
Close relatives	Close relatives
Social contacts	Social contacts
Time available	Time available
Work	
2. Personal characteristics	2. Personal characteristics
Mindset	Mindset
Personality	Personality
Comorbid symptoms	
3. Bipolar disorder	3. Bipolar disorder
(Hypo)manic symptoms	Hypomanic symptoms
Depressive symptoms	Stable mood
Stable mood	
Medication

**Table 3:** Themes and subthemes of facilitators and barriers during MBCT and corresponding quotations

	Facilitators	Barriers
1. Training		
Setting		
Location	*The location is easy accessible, both by car and public transport. I have a strong feeling, probably party due to my bipolar disorder, that in advance I worry too much about the accessibility (..) so bad accessibility can be an important reason for not joining the training *(16)	*The entrance of the hospital, also the smell, sometimes I am very sensible for this. That was what I felt, it was no aversion, but (..), when you go to the hospital, other associations take place (..) and that made it sometimes difficult for me* (5)
Space	*The location was nice; a quiet surrounding, a bright environment, sometimes they put some nice flowers in the room (..) and that felt good* (6)	*What I found difficult (..) I think interpersonal space is important to me, and with those kind of practices you need to feel safe in the room. And when you’re very close to each other, I don’t feel relaxed *(1)
Time	Frequency: *I thought the frequency of the training was good; once a week was fine for me. I think when the frequency would be once in 2 weeks, there would have been too much time between the sessions. For me personally, I benefit more from regular contacts (..) so for me it was important that the training had recurring sessions* (5)	Strict attendance: *It was possible to skip some of the sessions (..). Maybe it would have been more useful when we only could continue with the training when we participated in contiguous sessions. Right now it is too easy to continue when you miss some of the sessions. For me, a more strict approach is helpful to continue participation* (1)
Content		
Physical content	Silent day: *It wasn’t a big challenge for me to be quiet the whole day. Instead, I got the opportunity to attain a state of deep meditation as a result of the sequence of the practices. This made me think: “I would like to do this again, I think it is quite healthy”* (13)	Follow-up session: *I thought it would be nice if there would be another session after one month or so. Right now, the training was very intense and then it was suddenly finished.. You try to go on yourself but I thought it was a big transition* (3)
Materials	Audio tapes: *The audio tapes helped me; they made it possible to try another exercise every time (..). You have to try what suits you, and how you relate to it, and those tapes helped me with this *(13)	Homework forms: *The structure of those homework forms could be more clear. Right now, the answers could go anywhere* (3)
Homework	*Homework was another reason to reflect on the training. Besides, you have to practice with the things you have learned during the training (..). You are practicing to have more awareness, so I think doing your homework is an important part of this* (10)	*When I didn’t do my homework, the teachers did not seem to care (..). For me, it works better when homework is an obligation and they just say: “when you don’t do your homework, you have to stop with the training”. Homework is part of the training and when you don’t do this, there is no use to attend the course.. So, a more strict approach would be helpful* (1)
Teacher	*I think that it’s an added value that the teacher has a lot of experience with the limitations of the group—that the teacher has both knowledge of mindfulness and bipolar disorder, with regard to the symptoms and limitations of patients* (7)	*In my opinion the teacher paid too much attention to manic symptoms, which I didn’t like that much* (10)
Peer group	D*uring the training you are together with other people with a bipolar disorder; some of them a bit more hypomanic, others more depressive, so there is always someone you can relate to (..). Maybe I was longing for silence together with other people whose life is not always as easy*. (9)	*One person in the group was very present. When you gave her a little bit of attention, she wasn’t able to stop talking (..). I thought it was very annoying* (9)
2. Psychosocial		
Close relatives	*My wife encouraged me to do my homework every day. I have a tendency to forget things like this when I feel stable, she helped me to think about the homework so I could actually do it *(3)	*I recognize that it is difficult for me to keep on practicing at home. I think this is also because my family doesn’t have any interest in mindfulness and make a bit fun of it.. I think because of that, my enthusiasm dropped* (7)
Social contacts	*Many of my friends have experience with mindfulness. They all are healthy, but most of them have relational problems for which they followed a mindfulness training. They were very enthusiastic about it, which made me really enthusiastic as well* (10)	*I went to see my nurse and she asked me: “this mindfulness training, does it suite you?” I said: “no, I don’t think so”. Then she said:”No, I don’t believe it either. Shouldn’t you stop?” So I stopped *(11)
Time available	*I don’t have a job at the moment, so I have more time available then other people. This really gave me the opportunity to attend the training. Otherwise, you have to do the training between all other obligations* (2)	*I didn’t do my homework because I had too many other obligations which had priority* (1)
Work	Not applicable	*I was very busy with work, so during the meditation a lot of thoughts related to my job arose (..). It felt like a cyclone, it went faster and faster. This all happened when I was just lying down on the floor* (3)
3. Personal characteristics		
Mindset	Positive mindset: I* managed to keep on going because I had the conviction that the training would have lots of positive effects *(3)	Negative mindset:* I didn’t take the time during the week to practice (..), it took too much time. Actually, it doesn’t take that much time, but that is what I thought* (2)
Personality	Perseverance: *If I start something, I finish it. When I have started something, I will go every session and I will never cancel when I don’t feel like it* (9)	Impatience: *I am very impatient. I also feel like this with the audio recordings, like “come on, I know this already, hurry up” and then I press fast forward *(16)
Comorbid symptoms	Not applicable	Physical condition:* I have a bowel disorder (..) and you could imagine, every time you sit or lie down and you constantly have to stop the practice because you have to go to the toilet, doesn’t help. This is not only disturbing for me, but for everyone in the room *(4)
4. Bipolar disorder		
(Hypo)manic symptoms	*When I feel hypomanic I have more energy to really focus on the present, to be able to keep coming back to the present instead of drifting off and getting distracted all the time *(14)	*During the hypomanic period my mind was very busy and I didn’t feel the rest to attend the training *(1)
Depressive symptoms	Not applicable	*Difficulties with thinking, bad concentration (..), that caused a lot of difficulty for me to attend the training. When my energy level was really low I had thoughts like: “Do I really want this?*” (9)
Stable mood	*During the period of the training I had a stable mood. Because of this, I really enjoyed participating* (12)	*But yes.. the danger lies within the moment when you don’t really need it because your mood is stable (..), therefore I wasn’t motivated to practice* (6)
Medication	Not applicable	*Because of the medication I was less active and it was harder to feel my emotions* (7)

### (1) Training

For both facilitators and barriers this category was subdivided in four subthemes: *setting, content, teacher and peer group*. Setting was further subdivided in *location, space and time* and content aspects in *physical content, session, materials and homework*.

With regard to *location*, good accessibility was frequently mentioned as a facilitator. On the other hand, a location in or near the hospital was also perceived as a barrier as it reminded participants of earlier admissions. The teachers emphasized the importance of good accessibility and parking spaces as well.

With regard to the *space* the training was offered in, natural and quiet surroundings as well as the presence of materials acted as facilitators. Too little space in the room itself, being too close to other participants, lack of cleanliness and light were not considered helpful to attend the training.

Most participants thought the frequency of once a week was helpful regarding the subtheme *time.* The fixed training moments on the same day and time were appreciated by the participants. Some participants suggested to shorten the duration of the exercises. One participant experienced the silent day as too long, but most participants did not share this opinion and indicated that the length of the silent day was fine. The teachers indicated that they found it helpful to introduce a short break (of 10 min maximum) during the sessions.

Regarding the *content* of the training, all participants felt supported by the silent day. Session six, where partners or close relatives were invited, was generally considered as a facilitator, only one participant experienced it as too confronting. Other aspects which were experienced as facilitators were the breathing and walking meditation. Participants experienced the diversity of practices during the course as helpful, because this gave them the opportunity to be flexible and adapt the practice, depending on their mood. For example, when participants felt they had a hard time concentrating due to mood swings, they were able to choose shorter exercises. Lack of variety in the exercises and lack of follow-up meetings were experienced as barriers. The teachers confirmed that the partner session was facilitating. They indicated that is was helpful for participants to talk with their partner about the bipolar disorder, without feeling judged or without the presence of a direct trigger (e.g. acute mood symptoms).

Regarding the *materials,* most of the participants appreciated the presence of the workbook. Two participants, however, considered the folder as being quite unstructured. One participant had difficulties accessing the online audio files of the exercises, for others the online audio files were considered a facilitator.

Generally, *homework* as a part of the training was regarded as helpful, although most of the participants found the amount of homework too much. For some the self-imposed pressure to complete the homework worked as a barrier. For two participants the lack of strictness around the homework made it more difficult to actually do it.

Most patients felt supported by the *teacher* in terms of their high level of engagement; the pleasant, clear and soft voice; and their mild, open and calm attitude. It was also appreciated that the teacher was well informed about bipolar disorder. On the other hand, some participants reported lack of engagement and enthusiasm, an impersonal approach and judgmental attitude of the teacher as a barrier. Twice participants mentioned that the emphasis on (hypo)manic rather than depressive symptoms disturbed them. Another barrier was the absence of strictness of the teacher about homework and participation.

The fact that the training was offered in a *group* and stimulated peer support was brought up many times. The openness and safety experienced in the group was repeatedly mentioned as helpful. Two participants experienced the behavior of peers as disturbing. One participant mentioned that recognizing his problems in others was confronting. Too little personal attention from the teacher during the training because of the group setting was considered as a barrier. The teachers indicated the importance of the group size. They thought the group should not be larger than eight participants, because of the sensitivity for stimuli that some people with BD experience. Furthermore, they confirmed that peer support was important as well.

### (2) Psychosocial factors

Participants’ stories revealed four different subthemes*: close relatives, social contacts, time available* and *work.*

Attitude of participants’ family, brought together as *close relatives*, showed two different aspects experienced as facilitators, namely practical and mental support. Practical support consisted of taking over duties by family members so the participant had more time to attend the training and do his/her homework. Family members also played an important role in supporting the participant to remind him/her to do the homework. Mental support consisted of a non- judgmental attitude towards MBCT. On the contrary, a disinterest of family members made it harder for participants to participate.

Emotional support in the way of shared enthusiasm and positive experience with mindfulness of friends and acquaintances, known as *social contacts*, was only mentioned as a facilitator. Teachers mentioned that support from the attending clinicians is facilitating as well. In some cases the attending clinicians or nurses were not as supportive of the training, which clearly acted as a barrier.

Having *enough time* to participate was experienced as facilitating, while lack of time was repeatedly expressed as a barrier. Being preoccupied with other things, for example *work*, was a barrier. Also seeing work as a priority and have to work abroad resulting in the physical absence from the MBCT training were experienced as barriers. No facilitators in this subtheme arose.

### (3) Personal characteristics

Within this category three themes emerged: *mindset, personality and comorbid symptoms.*

Throughout the training a positive *mindset* made it easier for participants to attend the training. A positive mindset consisted of either a positive expectation of mindfulness or letting go of expectations. Previous experience with other kinds of meditation was regarded as helpful. Some participants mentioned a negative expectation of mindfulness as a barrier. Many participants did not prioritize mindfulness over other things in their life. Perceiving mindfulness as being isolated from the rest of their lives was also mentioned as a barrier. The teachers indicated that the expectations of mindfulness could act as a barrier as well. For example, one participant expected mindfulness to be about relaxation. When she found out this was not the case, and that there was expected quite a lot from her, she dropped out of the training.

Conscientiousness, curiosity, perseverance and taking care of oneself were facilitators regarding the subtheme *personality.* Within this subtheme, three different kind of subcategories arose: thinking, feelings, and perfectionism. The tendency of thinking rather than doing, the inability to concentrate and the repression of feelings, turned out to be barriers. One participant explained that he used the training as an escape to avoid feelings. Another barrier was the fear to disturb others during the training. Difficulties with taking time for themselves and relax, lack of perseverance and self-acceptance, and the feeling of inability to reach their own standard was repeatedly mentioned and made it more difficult for participants to attend the course.

One participant mentioned that the symptoms of his irritable bowel disorder, classified as *comorbid symptoms*, acted as a barrier throughout the course.

### (4) Bipolar disorder

Symptoms of the bipolar disorder were often mentioned by the participants. Remarkably*, (hypo)manic symptoms* could act as both facilitator and as barrier. For most participants (hypo)manic symptoms were experienced as a barrier to attend the training, to sit still and concentrate. This was due to having too much energy, being busy with other activities and finding it difficult to stop themselves. One participant actually experienced (hypo)manic symptoms to facilitate starting the practices due to the higher amount of energy. The teachers confirmed that (hypo)manic symptoms acted as a barrier, because participants were less likely to do their homework, were less likely to understand the content of the sessions, and disturbed the group process.

In contrast, *depressive symptoms* only acted as a barrier, namely in terms of lack of energy, concentration, motivation and focusing on the negative aspects of the training and homework. The teachers confirmed that depressive symptoms acted as a barrier, because it was harder for participants to stay motivated.

*Stable mood* could act as both as barrier and facilitator. For some, a stable mood acted as a facilitator to practice, because they were motivated to prevent relapse. For others, stable mood acted as a barrier, because they felt a lack of urgency to practice.

Difficulties to experience emotions as an adverse effect of *medication* was mentioned once as a barrier. Teachers mentioned that cognitive problems due to medication were a barrier as well, such as problems with focusing.

## Discussion

### Main findings

The aim of the present study was to look at the experience of adapted MBCT for people with BD regarding the facilitators and barriers of the training. We identified four key themes, concerning *training aspects*, *psychosocial factors*, *personal characteristics*, and *symptoms of bipolar disorder* itself. *Training* aspects involved setting, content, homework, teacher and peer group. *Psychosocial aspects* were found in close relatives, social contacts, and time available. The subtheme work within this theme was only found as a barrier. *Personal characteristics* appeared in mindset, personality and comorbid symptoms. *Bipolar disorder* was subdivided in (hypo)manic symptoms, depressive symptoms, stable mood and medication.

Unexpectedly, this study showed that people with BD regarded both the partner session (session six) and the silent day as a facilitator. The teachers expected the silent day would be too long for people with BD, because they experienced that people with BD often have problems with concentration and attention, independent of their mood state, which has been confirmed in literature as well (Wingo et al. 2009). Instead, participants report that they found the silent day facilitating, because it helped them to deepen their meditation practice and gain more insight in their behavioral patterns. This finding is in line with the study of Janssen et al. (Janssen 2017), that showed that a silent day was found helpful by people with ADHD as well.

Furthermore, our results indicate that depressive symptoms act as a barrier throughout the training, mostly because of the lack of energy, concentration and diminishing motivation. This is in line with the findings of Chadwick and colleagues (Chadwick et al. 2011), where a lack of motivation underlying depressive mood was found to be a barrier as well. Despite these findings, several studies showed that MBCT is effective in diminishing depressive symptoms in people with depressive disorder (van Aalderen et al. 2012; Strauss et al. 2014). This suggests that when people with BD experience depressive symptoms, they may need additional support (e.g. more contact hours, help with doing homework) to overcome this barrier, in which case MBCT could be effective in reducing their depressive symptoms.

Our findings suggest that the presence of current (hypo)manic symptoms can both act as facilitator and as barrier. This is not as surprising as it initially seemed, since (hypo)manic symptoms exist on a wide spectrum with varying levels of impairment and severity (American Psychiatric Association 2013). When people with BD are on the low end of the manic spectrum, during which they experience increased energy and focus but no functional impairment, this might help them to stay motivated to actually practice mindfulness. On the other hand, when people with BD are on the higher end of the manic spectrum and have difficulties concentrating, it might become harder to do the formal exercises.

Moreover, euthymic mood acts as a facilitator during MBCT, which is in line with the findings of Chadwick et al. (Chadwick et al. 2011). Besides functioning as a facilitator, we found that euthymic mood can act as a barrier as well, because participants feel less urgency to practice when they do not experience any mood symptoms. According to the study of Chadwick et al. (2011), the availability of different mindfulness practices was helpful, because participants could adapt their mindfulness practice according to their current mood state. This study replicates these findings, indicating that participants found it helpful to choose an appropriate practice according to their current mood state or available time.

## Strengths and limitations

The present study has several strengths. First, it took place in the context of a broader, well powered randomized controlled trial (Hanssen et al. 2019). Second, the training was taught by three different mindfulness teachers, together with three different nurses specialized in the treatment of BD, which makes these results more generalizable to other settings. The mindfulness teachers were interviewed as well to ensure triangulation of the data. Third, the research team was varied and consisted of six people with different levels of experience with MBCT and bipolar disorder, working in different mental health care settings, which ensured different perspectives on the data. Coding was carried out by two independent persons, ensuring the consistency of coding. The interviewing was also done by two independent and skilled residents in psychiatry, highly trained in questioning people with a BD. Data collection was not ended before saturation was reached and no new codes or information emerged from the data. To improve the validity of the study we conducted a member-check and included quotations. Fourth, participants were selected by means of purposive sampling. Participants who were considered as drop-outs were included in the study as well, which is important to consider possible barriers to participate in MBCT.

One important limitation is that it was not possible to interview people with BD who chose not to participate in the randomized controlled study itself. Information from these people may have been especially relevant to investigate possible barriers to participate in MBCT altogether, such as particular preconceptions about mindfulness.

## Implications for clinical practice and research

Implications from the current data for clinical practice include different aspects. At first, we might consider to offer MBCT in a neutral training setting, because a hospital/psychiatric clinic might be too confronting due to previous admissions. Secondly, the partner session (session six), the silent day, and the time and content of the training were regarded as helpful, suggesting the original protocol of MBCT might only need slight adaptions made for BD. Thirdly, additional support (e.g. more contact hours) according to the current mood state might be necessary to help participants to stay motivated to complete the training. Furthermore, teachers might need to be more flexible towards the use of formal practices, to help participants to adapt the practices to their current mood states. For example, in case of depressive symptoms it might be helpful to support participants to use more active formal exercises (e.g. walking meditation). In case of (hypo)manic symptoms, it might be helpful for participants to focus more on the body instead of their racing thoughts (e.g. body scan). A personal approach of the teachers towards the participants is necessary to adapt the training and formal exercises when necessary. Finally, it might be important that MBCT for people with BD is conducted by a pair of teachers. This might be helpful to enable a more tailored approach towards the participants, for example by one teacher being able to take a participant who feels overwhelmed apart, while the second teacher tends to the group.

The present qualitative study generates hypotheses and possible directions for future research. However, it does not quantify the effects that were mentioned by participants. Considering future directions of research, more data on the effectiveness of MBCT from adequately powered, randomized controlled research are needed (Hanssen et al. 2019).

## Conclusion

The present study shows that MBCT, when taught by qualified mindfulness teachers following the original MBCT program with minor adaptions, is a feasible intervention for people with BD. Important clinical implications are suggested, including the need for additional support and a flexible approach from the teachers depending on the current mood state of participants. Future quantitative studies are necessary to determine whether MBCT is a (cost-)effective treatment for people with BD.

## Data Availability

The datasets used and analysed during the current study are available from the corresponding author on reasonable request.
